# “He Never Wants to Leave. I Would Leave in a Second.” Examining Perceptions of Rural Life and its Impact on Families who Migrate for Employment and Those who Stay Behind in Atlantic Canadian Communities

**DOI:** 10.3389/fsoc.2021.578647

**Published:** 2021-02-12

**Authors:** Christina Murray, Hannah S. Skelding, Doug Lionais

**Affiliations:** ^1^ Faculty of Nursing, University of Prince Edward Island, Charlottetown, PEI, Canada; ^2^ Master of Global Affairs, Universidad Rey Juan Carlos, Mostoles, Spain; ^3^ Shannon School of Business, Cape Breton University, Sydney, NS, Canada

**Keywords:** family, mobile work, rural community, gender, Atlantic Canada

## Abstract

In Canada, interprovincial labor migration is a common form of mobile work that is significant for rural communities especially in Atlantic Canada. Unique to this form of labor migration is the gendered nature of the phenomenon resulting in men often leaving their wives, families and rural communities behind for employment opportunities in the oil and gas sectors thousands of kilometers away. As men leave their families and communities for employment, women who are left behind become primary caregivers to children in addition to also being the primary caretakers of the family home. The Tale of Two Islands project was a multi-year, cross regional mixed methods research study that examined labor mobility and its impact on families and communities. This paper examines how labor migration has impacted families and rural communities. Drawing upon focus group, conversational and key informant interviews with families impacted by mobile labor and practitioners who serve them, societal perceptions of gendered norms and perceptions of rural life became illuminated. This has contributed to multiple contradictions and role confusion as families adapt and adjust to periods of reunification and separation while striving to remain connected to their rural communities. Men yearn for opportunities to be an active member of their home communities but cannot a result of living away for extended periods of time. In contrast, women who stay behind in rural communities often chose to isolate from community activities as a result of perceived judgments that are attributed to traditional views of rural life and family roles.

## Introduction

Canada consists of many communities experiencing the phenomenon of men leaving their families behind in pursuit of employment opportunities within the country. This unique form of migration is often referred to as interprovincial labor migration (Morse and Mudgett, 2018). Many of the people that engage in interprovincial labor migration from Atlantic Canada are men with wives and families who travel for employment while maintaining homes and supporting families left behind. For men who fly long distances for employment, work patterns may be predictable with predetermined schedules known or they may be highly unpredictable and return to home dates uncertain. Whether known or unknown, repetitive patterns of separation and reunification, contribute to family disruption and disorganization (Parreñas, 2000; Grzywacz et al., 2006; Taylor and Graetz Simmonds, 2009; Torkington et al., 2011; Wray, 2012; Meredith et al., 2014; Murray, 2014; Vodden and Hall, 2016; Donatelli et al., 2017; Murray, 2017; Scannell and Gifford, 2017; LeDrew et al., 2018; Neil and Neis, 2020). When men in families leave their homes and communities behind for employment opportunities, it is often their wives who remain behind and take on the primary responsibilities of the house and family that would normally be shared with their partner. The long physical distances associated with migrant work impact the worker’s relationships with their spouse, children, and extended family. Along with a change in family dynamics comes a shift in the fabric of the community. Men who work away from their “home communities” are not present for extended periods of time to experience the day-to-day functioning of their family within the context of rural community life. There can be vast differences in the perceptions of community and rural life between men who are working away and women who remain behind in the community. Therefore, it is critical to examine the familial perceptions of interprovincial labor migration and rural community life through a gendered lens.

In Canada, the phenomenon of interprovincial labor migration has been most apparent in the four provinces of Prince Edward Island, Newfoundland and Labrador, Nova Scotia, and New Brunswick. As a result of Canada’s Freedom of Mobility Act statistics regarding the total number of people leaving Atlantic Canada for interprovincial labor migration are not known (Murray, 2014). However, within the communities in Atlantic Canada it is well known that many men are engaging in this form of mobile work as a result of the economic instability in the region (Lionais et al., 2020). Specifically, within the Atlantic region, P.E.I. and Cape Breton Island are two areas that have been significantly impacted by interprovincial labor migration. Both P.E.I. and Cape Breton have a significant number of men engaging in this form of migrant work. P.E.I. is the smallest province in Canada, both geographically and by population. There are approximately 157,000 people currently living on P.E.I. (P.E.I. Statistics Bureau, 2019), with an estimated 51,000 people living in two identified cities, Charlottetown and Summerside (Prince Edward Island Department of Finance, 2019). This is significant when compared to the Canadian population as a whole, whereby 71.5% of the current population lives in an urban metropolitan area (Statistics Canada, 2019). Nova Scotia shares similar characteristics to P.E.I. Between 2015 and 2016, the total estimated population of Nova Scotia had a 0.6 percent increase (Nova Scotia Finance and Treasury Board, 2017). However, during that time only the urban Halifax County and three other counties (Annapolis, Kings, and Antigonish) increased in population (Nova Scotia Finance and Treasury Board, 2017). Notably, Cape Breton County experienced the largest population decline between 2015 and 2016 (Nova Scotia Finance and Treasury Board, 2017). This population decline in Cape Breton has a long history. Between 1991 and 2006, 37.7% of the population aged between 20 and 34 left the area (Wray, 2012). While Cape Breton makes up approximately 15 percent of Nova Scotia’s population, from 2006 to 2011, 41 to 45 percent of all workers engaging in interprovincial labor migration from Nova Scotia to Alberta were from Cape Breton and in 2011, employees from Cape Breton Island represented 5.5 percent of Alberta’s employed labor force (Lionais et al., 2020). This finding shows the significant impact interprovincial labor mobility is having on rural populations and migrant labor flows from rural Atlantic Canada.

The primary economic drivers in P.E.I. and Cape Breton rural communities are seasonally based industries, such as fishing, farming and tourism (Lionais et al., 2020). Seasonally dependent industries offer limited employment and economic opportunities. Individuals who are employed in seasonal sectors often have higher rates of unemployment, decreased individual and family incomes, lower levels of educational attainment, and increased rates of out-migration (Wray, 2012; Statistics Canada, 2015; Hardy et al., 2018; Prince Edward Island Department of Finance, 2019). As of January 2020, the overall unemployment rate in Canada was 5.5 percent (Statistics Canada, 2020). However, in P.E.I. and Cape Breton the unemployment rates were 8.8 percent and 12.2 percent respectively (Statistics Canada, 2020). The consequences of seasonal employment and subsequent unemployment lead to people relying on Federal Government employment insurance benefits as a source of income supplementation. Federal benefits are frequently less than half of one’s income earned when employed and contribute to precarious financial situations for individuals and their families. It is clear that family finances act as a catalyst behind a person’s decision to leave their family and rural community in search of better employment and economic opportunities.

There is a reciprocal interconnectivity with mobile families impacting rural communities and simultaneously rural communities impacting mobile families. Not only has the oil and gas sector in Alberta impacted P.E.I. and Cape Breton from a social perspective, it has also shaped these communities economically. In 2011 the income earned in Atlantic Canada by interprovincial employees added 2.3 billion dollars to the Atlantic Canadian economy (Lionais et al., 2020). From this total income, interprovincial employees working in Alberta contributed one billion dollars (Lionais et al., 2020). Forty-five percent of interprovincial employment income in P.E.I. was from Alberta (Lionais et al., 2020). In Cape Breton, 64.8 percent of the income generated interprovincial employees annually came from Alberta (Lionais et al., 2020). This economic inflow into P.E.I. and Cape Breton reveals the significance of interprovincial employment for rural communities in these provinces. It also illustrates how dependent rural communities have become on remittances earned from interprovincial employment as a key economic source to support rural community sustainability in Atlantic Canada. With such a small population base and high reliance on oil and gas remittances as economic generators have recently been quantified however, the personal and family impacts of interprovincial labor migration remain often overlooked.

Mobile work opportunities, such as long-distance commuting in the mining and oil and gas sectors, create a distance between workers and the rural community (Meredith et al., 2014; Vodden and Hall, 2016; Dorow and Mandizadza, 2018; Morse and Mudgett, 2018). Life in rural areas is complicated through the considerations of movement, mobility and place attachment between workers and their rural communities (Scannell and Gifford, 2017; Morse and Mudgett, 2018). Within Atlantic Canada, Prince Edward Island (P.E.I.) and Cape Breton Island, Nova Scotia, are islands comprised of small, rural, and often isolated communities. These communities are defined by close-knit relationships and may be representative of multi-generational families who have deep roots in the community often spanning back to Canadian confederation. Being rooted in one’s community contributes to a sense of community ownership and wellbeing. This holds especially true for mobile families living in P.E.I. and Cape Breton who identify their rural Island communities to be their “home community” even though their physical presence in these communities is limited due to their out of province employment. This sentiment is associated with a belief that living in a small community contributes to a sense of well-being and is positively associated with quality of life for both individuals and families (Scannell and Gifford, 2017). As mobile workers continue to work away from home their attachment to their community is continually disrupted.

## Background and Context

Labor migration is a global phenomenon. The United Nations International Office of Migration recently reported that in 2020 there were 164 million migrant workers. These workers provided 689 billion US dollars in remittances (McAuliffe et al., 2019). From review of literature, it has become evident that there is a lack of consistency in terminology to describe the type of employment where mobile workers are employed within their country of residency but working in locations thousands of kilometers away from their families and home communities. The following terms have been identified and used interchangeably in literature to describe this form or labor mobility: fly-in-fly-out, drive-in-drive-out, long distance commuting, commute work, long distance labour commuting, extended commuting, circular migration, circular mobility, commuting work, long-distance commute work, job-related spatial mobility, pendulum migration, seasonal migration, oscillating migration, geographic mobility. Additional terms such as temporary foreign workers, guestworkers, and non-resident employment have also been used to describe employment patterns when employees travel out of country for employment. This lack of consistency in terminology contributes to a global lack of understanding, programs, policies and practices that specifically address this unique form of employment mobility.

Additionally, there is a sparsity of research conducted specifically on how travelling long distances for employment impacts families and home communities. Delving further into literature examples from Australia, the Philippines, and Mexico were discovered. In these countries employment occurred internally or internationally resulting in prolonged periods of family separation (Parreñas, 2000; Grzywacz et al., 2006; Taylor and Graetz Simmonds, 2009; Torkington et al., 2011; Meredith et al., 2014; McAuliffe et al., 2019). Australian researchers reported that among fly-in and fly-out workers there are higher rates of marital stress, divorce and extramarital affairs (Torkington et al., 2011). Additional studies revealed that fly-in and fly-out employment, was disruptive and affected the psychological wellbeing of families (Grzywacz et al., 2006; Torkington et al., 2011; Meredith et al., 2014). In the Philippines, the majority of migrant work is completed by women who are often employed for defined periods of time as caregivers in other countries. Women often leave their children and partners behind for these employment opportunities (Parreñas, 2000; Parreñas, 2001). In the Philippines, to leave one’s country for temporary work in another, is lucrative and highly favored as a middle-class professional stream due to the significant remittances that are returned to their families (Parreñas, 2000). However, women who engage in mobile work can encounter discrimination which negatively impacts their psychological well-being (Hall et al., 2019). In the United States, approximately 75 percent of migrant farmworkers come from Mexico (Mccoy et al., 2016). Researchers have discovered that depression, anxiety, substance abuse, and high-risk sexual activity are experienced more frequently among these farmworkers (Torkington et al., 2011; Mccoy et al., 2016; McAuliffe et al., 2019).

Upon further examination of literature, it was noted that the experiences of families impacted by mobile labor are deeply rooted in gender. Globally trends in migrant labor also reveal the gendered nature of this phenomenon with 96 million men compared to 68 million women employed as migrant laborers in 2017 (McAuliffe et al., 2019). In the Philippines women working abroad continue to be the primary caregiver and authority who is making decisions for their children while they are working away (Parreñas, 2000; Parreñas, 2001). They also provide emotional support for their husband (Parreñas, 2000; Dorow and Mandizadza, 2018). When men are the migrant workers the roles associated with gender do not change, women still hold onto a larger share of household and familial responsibility (Dorow and Mandizadza, 2018). Similar to Australia, interprovincial labor migration in the Canadian context is dominated by men, with at least half of those working in the oil and gas sector being men (Wray, 2012; Dorow and Mandizadza, 2018; Neil and Neis, 2020).

Unlike, guest workers, or temporary foreign workers, Canadians commuting long distances for work are citizens and can travel without documentation or restrictions throughout the country. They can retain residence in one province yet travel frequently to locations thousands of kilometers away for employment. Skeldon (Skeldon, 2012) identifies internal labor migration to be the most ideal form of labor migration because internal migrants, in most countries, are free to come and go as they please, unrestricted by the laws and employment policies associated with entering and working in a foreign country (Skeldon, 2012).

Due to the large geographical size of Canada, Atlantic Canadians workers must fly in and fly out for employment in the oil and gas sectors of Western Canada. Unlike Australia very little is known about the experiences of Canadian workers and their families who are impacted by interprovincial labor mobility. While Australia has formalized services, supports and policies specific to the needs of fly-in fly-out families (Meredith et al., 2014), Canadian policies and supports specific to the needs of families impacted by labor mobility are lacking. It has only been within the past 10–15 years that programs of research specific to interprovincial labor migration and the experiences of families has emerged (Wray 2012; Murray, 2014; Donatelli et al., 2017; Dorow and Mandizadza, 2018; LeDrew et al., 2018; Neil and Neis, 2020). The lead author of this paper was one of the first Canadian researchers to begin a program of research exploring family experiences of labor migration and its impact on rural communities in Atlantic Canada. Beginning with her doctoral study focusing on the experiences of women left behind, to the intergenerational family experiences of labor migration, to the first ever Canadian symposium on families, work and mobility, her program of research has helped to advance understanding of this issue in Canada.

Atlantic Canada has the highest proportion of people living in rural communities and is characterized by having older populations higher rates of seasonally dependent employment, unemployment, and poverty (Stattistics Canada, 2015; Hardy et al., 2018; Statistics Canada 2018). Rural community and identity are important factors to consider when examining the impact of migration and the push-pull factors that contribute to men deciding to leave their families behind for employment. Motives for out-migration from rural spaces are drastically different from the motivations for leaving an urban area. Factors such as employment, education, sense of belonging, and family support have a large role in the decision-making process of individuals who choose to leave their rural community (Morse and Mudgett, 2018; Neil and Neis, 2020). There is a dominant cultural construction of rurality that is strongly connected to stability, rootedness, and attachment to place (Milbourne and Kitchen, 2014; Morse and Mudgett, 2018). This notion of rurality implies that rural spaces are not as prone to mobility as urban spaces. In comparison to urban spaces, it is a common narrative to assume that rural spaces are less mobile than urban (Milbourne and Kitchen, 2014). People choose to live in rural areas for many reasons (Morse and Mudgett, 2018). At the forefront of these decisions is a strong connection to place and family (Morse and Mudgett, 2018). For many people, staying in a rural place is an active decision (Morse and Mudgett, 2018). While there is a perceived nature of stability associated with rural communities, these spaces also experience a complex range of mobilities (Milbourne and Kitchen, 2014).

In Canada, interprovincial labor migration impacts not only the worker, but also their family who remains behind in the rural community (Wray, 2012; Markey et al., 2015; Vodden and Hall, 2016). The nature of interprovincial labor migration in Canada mimics the societal standards that are commonplace in Canada, especially in rural areas (Dorow and Mandizadza, 2018). Regardless, of employment location and actual amount of time spent with their families, men consider their rural communities to be their home communities. They have a strong sense of place attachment. In their absence, wives adopt additional roles and responsibilities as they become the primary caregiver to children, and caretaker of homes, in their partners absence (Murray, 2014; Dorow and Mandizadza, 2018; LeDrew et al., 2018). These experiences can shape perceptions of rural community life differently depending on whether one is leaving for employment or staying behind. To understand this more fully, it is critical to examine both of these perspectives.

## Methods

Examining the phenomenon of interprovincial labor migration in Canada to date, it has become apparent that a gap in knowledge specific to understanding the experiences of those who leave for work, family members who are left behind, and the resulting impact on rural communities exists. This labor migration trend is particularly relevant in the Atlantic Canadian provinces, where high numbers of laborers are working outside of their home province. Due to the Freedom of Mobility Act, specific statistics are not known, nor kept, regarding the actual percentage of people leaving the Atlantic Region on a temporary basis for employment in another province (Murray, 2014). The lack of data specific to temporary inter-provincial employment patterns is problematic, as it is difficult to gauge how many people are claiming permanent residency in one province yet is employed on a temporary or full-time basis in another. Without having measures in place to quantify population trends specific to temporary labor migration, there exists a gap in understanding the magnitude of this phenomenon. Further understanding how families and communities are impacted is near impossible. However, this information is vital if we are to find strategies to support individuals, families, and communities affected by temporary labor migration. While there has been research conducted regarding labor migration and how it was experienced by men who left for employment out of province in Cape Breton (Wray, 2012; Dorow and Mandizadza, 2018) and women who were left behind and living in Prince Edward Island (Murray, 2014), there remained a gap in research specific to the family experiences of labor migration from both an interdisciplinary and cross-regional, Maritime Canadian perspective.

To address this knowledge gap, the research team engaged in a multi-faceted, three-year research study examining the familial and community impacts of labor migration. Titled The Tale of Two Islands, this was to our knowledge, the first Canadian study to examine interprovincial labor migration and its impact on intergenerational family members, and rural communities. The objectives of this study were to; compare and contrast how temporary labor migration was being experienced by those who leave for employment, their partners who are left behind and caring for children, and their extended family which may include grandparents, brothers and/or sisters who provide a supportive role to the family; to compare and contrast how the temporary labor migration of family members influenced perceptions of family roles, family cohesion, and evolution by those who are leaving for employment and those who are left behind; and, to, elicit an intersectoral perspective regarding how labor migration to the oil and gas sectors of Northern Alberta shaped family evolution and community development across Prince Edward Island and Cape Breton Island.

To achieve our goal and objectives of this study, an interdisciplinary research team from the Faculties of Nursing and Business across from two academic institutions (University of Prince Edward Island and Cape Breton University) in two Atlantic Canadian provinces (Prince Edward Island and Nova Scotia) engaged in a wide array of data collection methods across our two provinces including a systematic literature review and quantitative analysis of oil and gas income remittances and the impact of these on the Atlantic Canadian economy (Donatelli et al., 2017; Lionais et al., 2020). Using a form of narrative inquiry research (Clandinin and Connelly, 2000; Connelly and Clandinin, 2006), the research team also engaged in conversational intergenerational family interviews (N33) with men who traveled for employment, their wives who remained in the community and cared for their children and extended family members such as grandparents who provided a supportive role; semi-structured key informant interviews with community-based professionals (Donatelli et al., 2017) who worked directly with mobile families (N24); and also conducted three focus group interviews (N24); one with women whose husbands worked out of province, and two with grandparents (grandmothers and grandfathers) who lived in the family’s rural community and provided support to their families who had a loved one working away. Each phase of qualitative data collection; conversational family interviews, semi-structured key informant interviews, and focus group interviews, occurred simultaneously across our two Islands to discover similarities and differences in how family experiences and community impacts. Ethical approval for this study was obtained from the University of Prince Edward Island and Cape Breton University.

Dewey’s theory of experience was a central theoretical underpinning that guided the qualitative aspects of our research and is central to how narrative inquiry has been conceptualized by Clandinin and Connelly (Clandinin and Connelly, 2000). Dewey views experience to be a continuous interaction of human thought within our personal, social, and material environment resulting in experiences that continually occur over time and are recalled in light of present and future experiences (Dewey, 1986). Data collection instruments developed for the study aligned with Dewey’s theory of experience with questions asked illuminating time; past, present and future; personal and social relationships; those between family and community members, and environment; that being life in rural communities. Dewey’s theory of experience was also central to how the project team approached thematic analysis. The goal of narrative inquiry research is to increase understanding and/or explain the meaning and significance of experiences through the sharing of stories. The process of telling and listening to stories is common to the human experience of being in the world and relating with others. Storytelling holds a cultural significance in Cape Breton as collective family narratives illuminate ancestral historical evolution, and how families have been shaped by their place within the community (Corbin and Rolls, 1996). The importance of storytelling is also apparent within the context of family evolution and development in Prince Edward Island.

All interviews were digitally recorded and transcribed verbatim. NVIVO software was used for initial data analysis with codes and themes identified. Further analysis occurred through multiple team meetings where the team examined multiple narratives illuminated as they pertained to roles and relationships among family members and community, temporarily of experiences - past, present and future and community impacts and interconnections. As intergenerational family members shared their stories of experience regarding how labor migration impacted their families, we also gained an increased understanding and awareness regarding how labor migration impacted rural communities in Atlantic Canada. This paper weaves together varied experiences and narratives that reveal how the coming and going for employment in the oil and gas sectors in Northern Alberta impacted family and community life in Prince Edward Island and Cape Breton Islands.

Findings of this research revealed how family members and professionals who work with families in Prince Edward Island and Cape Breton Island view labor migration to impact individuals, households, and communities. Drawing upon conversational intergenerational family interviews and key informant interviews we will share how participants conceptualize their rural communities and rural life. We will highlight how these perceptions vary significantly between those who leave the community for employment and those who are left behind. Particularly, we will illuminate the interconnectedness of place to family evolution in light of contrasting societal perceptions of gendered and familial norms. We will also present how these perceptions have contributed to feelings of being negatively judged by others in the community which has contributed to increased social isolation and a decreased sense of belonging, which has impacted their families’ participation in community activities.

## Results

During multiple individual conversational, key informant and focus group interviews, participants shared their stories of experience related to interprovincial labor migration. These experiences illuminated how men and women as husbands and wives conceptualized their experiences of mobile work and how this impacted role functioning, family development and their perceptions of community and rural life. Various practitioners interviewed across P.E.I and Cape Breton Island identified the many challenges and difficulties experienced by families impacted by interprovincial labor migration. These were often attributed to perpetual periods of family separation and reunification. The stability of a family unit that permanently lives together day in and day is gone, replaced by a certain uncertainty that is associated with interprovincial labor mobility. These families experience first-hand how their community engagement differs from other families living in the community not involved in interprovincial migration. The state of mobility that these families experience often generates barriers and obstacles for them to participate in various events and volunteer activities occurring in their local community.

Analysis of our data revealed that men and women had differing opinions in regard to their sense of place attachment to their rural home communities. All men and women interviewed considered at least once, permanently moving to Western Canada so that their families could be geographically closer together in hopes of seeing each other more frequently. Men participating in our study reported that they lived less than 25% of the year with their families and in these Island communities, however in spite of their frequent absence, they felt a strong sense of place and always referred to these communities as home. Men were rooted in their communities and could not imagine their families moving away. They spoke of Alberta being the place where they worked whereas their PEI or Cape Breton communities was home. As one participant explained; “*We weighed the pros and cons and it always comes down to staying. We have talked about it, but when we actually sit down and weigh the pros and cons, it’s just not worth it. It’s easier for me to go than it is for everyone (the family) to go. At the end of the day, I am still going to be working 2 weeks on and 1 week off or 3 weeks on and 1-week shift and I’m going to be in a workcamp. When I think about raising my own family, I didn’t want to do it in Alberta. I wanted to do it at home. Unless we are forced into it, we absolutely do not want to move out there. Our families are here, my favorite people are here. This community is where we live, it’ a part of us. My parents live down the road from us, not far at all. That is why we are here. We are not going anywhere.”*

Men interviewed also spoke about the sense of safety and security that they felt having their families living in a rural community. They identified the increased prevalence of illegal drug and alcohol use, crime, lack of social support and the high cost of living as additional reasons why they would not want their families moving to Alberta. In contrast to these views, women participating in our study, expressed a yearning to move away from PEI and Cape Breton so that their families could be together more often and develop as a unit without the iterative patterns of separation and reunification. Reasons for this included being able to escape perceived judgment from others living in the community, having an opportunity to be physically and emotionally closer to their spouse, and having their children to spend more time with their father. As one woman revealed, *“He is a country boy, and we live on the same road as his parents and where he grew up. He’s not having his kids grow up anywhere but here. He never wants to leave. I would leave in a second. I don’t like living in a small community at all.”*

Many of the participants in our study described life in their rural communities to be both a positive and negative experience for their families. As one participant phrased it *“[y]ou know everybody which is good in a way and not in others.”* The dynamics of rural community is a balance. One characteristic that draws people to live in a rural area is the sense of community that comes from knowing the members of your community and also being known by them in return. However, a downfall of a close-knit community is the judgment cast when everyone knows each other and knows what is happening throughout the community. Without connections firmly established, it is easy to feel excluded in rural communities. Creating these connections is especially challenging for mobile families. As a Church Minister shared during a key informant interview, *“things take time, relationships with small communities, they have to trust you and you have to build it up.”* This statement frames what is necessary in order to build community in rural spaces. It requires time and energy to build connections and a sense of community within a rural area; something frequently lacking when a member of the community is present so infrequently.

The nature of interprovincial labor migration detracts from the time and energy that mobile families possess. When men return home, they are less likely to engage in their community as they are focused on making up for lost time with their family. While their partners work away women take on the role and responsibilities of their partner in addition to their own role as a participant explained; *“Most people live a life where every day is relatively the same. For people like us who do this, it’s like living two lives. You become a Jill of all trades.”*

Men who are working away feel distant from their local community due to the physical distance between their work and their home. This can complicate communications with family and friends back home, especially when something unexpected occurs. A participant explained; “When you’re out there working and something happens to someone or goes on at home, someone gets ill, it’s tough on a man. You’re far away, and you’re wondering what to do.” Work in Western Canada is accompanied by a three to four-hour time difference. For some families, the solution to the time difference is that they need to schedule phone calls and other forms of communication. This structured form of communication can cause a feeling of disconnection. The time difference also adds to the traveling time to come home. Jetlag detracts from the time the husband gets to spend at home in the community. This perpetuates feelings of distance. Additionally, the areas where these men are working often do not have adequate technology to maintain consistent communication with their families at home (LeDrew et al., 2018). This can result in men not receiving regular updates regarding the happenings in their families and home communities.

Many men in our study identified that if they were not away for work, they would be actively volunteering in their communities. Volunteer fire fighting, coaching sports, and engaging in a local church are all forms of volunteering that these men described missing out on. A participant explained; *“There’s no opportunity or time to volunteer or get involved with a lot of things … you don’t get the chance to consistently be there for the community … you get less opportunity to be with people in your community, even your neighbors.”* Due to increased familial responsibilities women whose husbands work away often do not have the time to participate in their communities. A key informant practitioner explained; *“women are so busy with their children they don’t participate in community events.”*

Men who participated in the study also shared that the longer they participated in interprovincial labor mobility, the less connected they felt to their home communities. A participant described this by saying, “*I work away and haven’t been here much for three and a half years. It feels like home to me, but it doesn’t feel like I’m part of the community.”* This is an important acknowledgment as rural identity is connected to a sense of closeness within your community. Another man reflected on a conversation he had when he was at home when a had friend mentioned his neighbors whom he did not know. This would be very unusual for members of this community not to know one another. He shared; *“They are just two houses down the road, and I don’t know who they are. I’m never here so I’m not in the loop.”*

Women shared that they felt that they were being judged by others in the community because their husbands were employed in Western Canada. They discussed at length comments that they had heard from community members regarding how wealthy their families were because their husbands worked out of province and how they chose this lifestyle so that they would be better off financially than the rest of the community. A participant revealed; *“I always get comments about how he is bringing home the big bucks That’s all they talk about as soon as I go anywhere. I don’t like it. I don’t like going to the store because I know as soon as I walk out someone is going to be talking about me.Everyone makes everyone feel insecure about this.”* In contrast her husband dismissed this to simple gossip and felt that; *“with every community there is gossip and it’s just the way it is here.”* From his perspective the gossip is not targeting his wife as a result of his work, but rather he attributed it to living in a small community.

Women also felt judgment from others in their home communities regarding the strength of their relationship with their husband. Since their husband is gone for long periods of time there was speculation by some community members regarding the stability of their marital relationships. Participants identified that they chose to attend an adult social event, such as a dance, by themselves, it could be interpreted by members of the community as an attempt to begin an extramarital affair. The fear of this judgment was frequently cited as a reason why women did not feel comfortable participating in community activities while their husbands were working away. It also contributed to increased feelings of stress, anxiety, shame and embarrassment which took a toll on women’s mental health. A social worker explained during a key informant interview*, “I think these folks are overrepresented in community mental health … you’ve got really worn-out moms who are stressed out and wanting to go off work or visiting their doctor more often because they can’t cope.”* The differing gendered experience was reinforced by men who did not report experiencing feelings of judgment by others in their home communities because they worked out of province.

Continuing along this theme, some women participating in our study felt that they did not “fit in” with the community when their husband was working away. One participant shared that when her husband is away, she does not like to engage in social activities alone because it makes her “stand out.” This concept of standing out is an interesting one. There was a shared sentiment among these women that because they are married, yet often live apart from their husbands, that they are different from other women and families in their community who have their husbands living with them in their home communities. The feelings of difference that these women perceive may be exaggerated by the nature of rural life where communities abide by commonalities of shared experiences (Haugen and Brandth, 2015). If someone no longer aligns with the societal norms of the community, they are “othered” (Haugen and Brandth, 2015). Women perceived that they and their family form was different and in turn, felt othered by their community. They felt alone in their experience and even though other women in their social circles also had husbands who were working away. This perception was illuminated by a participant who shared that she felt as though she was one whose husband worked in Western Canada. In contrast, her husband noticed a stark absence of fathers at their daughter’s birthday party; *“there were seven mothers there, no fathers. They are all working out west. Seven.”*

## Discussion

The historically weak economies in Atlantic Canada have led to a longstanding relationship between rural communities and mobile work. During our research we discovered multiple contradictory examples of how life in rural communities is perceived to be different by husbands who leave for work and their wives who remain behind. Husbands viewed their wives to be enveloped by communities of support, where they are surrounded by other women who also have a loved one traveling out of province for work. Men tend to view community gossip as a function of rural life, not as a result of the mobile nature of his work. This presents an interesting insight into the dichotomy of perceptions between husbands and wives equally impacted by the same phenomenon of mobile labor. Contrastingly, their wives perceived that they bear the brunt of community gossip and judgment as a direct result of their husbands work in Western Canada.

It is challenging to estimate exactly how many families are engaged in interprovincial labor migration however, one teacher who participated in a key informant interview, estimated that 15 to 20 percent of children attending their school had a father who was working out of province. While the impact of interprovincial labor mobility on children was not within the scope of this particular study, all participants spoke about the ways in which they noticed this phenomenon impacting children left behind. The notion of prevalence in rural PEI and Cape Breton communities was also apparent among other participants during key informant interviews. These practitioners estimated that one out of every four or five families living in their rural communities were impacted by interprovincial labor mobility.

Projections such as these, while not substantiated, could translate into a large portion of families who are living in rural communities and experiencing additional challenges and stressors as a result of having a loved one employed out of province. With this in mind, it is critical for communities to begin to recognize how having a loved one working away impacts families and develop supportive interventions that are responsive to their unique needs. Having community support that is perceived as welcoming and not judgmental has the possibility to change the experiences of mobile families. Through enhanced programming and targeted interventions, families could meet other families also living in the community and develop increased support networks that could reduce feeling of loneliness, isolation. This could lead to greater investment and engagement in activities occurring in rural communities.

Both husbands and wives acknowledge a disconnect from their home community. The husband’s perspective of this difference is grounded in perceptions of physical distance. His distance from the community is viewed as a result of his absence from their home community. However, women attributed their disconnect lack of time due to their increased family roles and responsibilities and perceptions of judgment by others in the community. However, it is important to acknowledge that families are not the only party negatively impacted by interprovincial labor migration. The community as a whole is disadvantaged through the exclusion of these families. For example, male role models are lacking in the community. A participant suggested that an increased emphasis needs to be placed on bolstering local economies so that that families do not need to live apart. They identified that, “it would be nice to have people working in the community and prospering in the community. People would be proud of that.”

## Conclusion

The Tale of Two Islands project offers an example of how mobile work impacts families and communities in Atlantic Canada. While this form of labor has positively benefited the economies of Atlantic Canadian communities, it has come at a social cost. Throughout this paper we have examined how interprovincial labor migration impacts mobile families and the rural communities they call home. Perceptions of interprovincial labor migration differed between the men that travel for employment in Western Canada and their wives who remained behind in their rural Island home communities. Men who engage in this work are not physically present in their family units or communities. While working away, men felt that they were missing out on an opportunity to contribute to their rural communities such as volunteering in community organizations or in activities where they could provide leadership and be a role model to others. In contrast, wives who remain behind and live in the rural community while their husbands work away are physically present and could participate in community activities yet are reluctant to as a result of increased familial roles, responsibilities and perceived judgements from community members. These are attributed to societal pressures and traditional views of gendered norms associated to men, women and families who live in rural communities (Wathen and Harris, 2007; Leipert and George, 2008; Haugen and Brandth, 2015; LeDrew et al., 2018).

Communities and families impacted by interprovincial labor migration are constantly shifting and changing, responding and adapting, in order to adapt to a mobile lifestyle characterized by periods of reunification and separation. While this paper has illuminated contrasting views among men who travel for work and their wives that remain behind in their home communities, these families have chosen to remain in their rural community as they embark in a mobile lifestyle. This decision is largely based on a desire to belong and have a sense of connectedness to their communities. Ultimately, the outcomes of their decision to remain rooted in their community are inherently gendered. Therefore, changes surrounding mobile work in rural communities need to be addressed in light of the impact gender roles have on the experiences of mobile families in rural spaces.

## Data Availability

The raw data supporting the conclusions of this article will be made available by the authors, without undue reservation.

## References

[B1] Clandinin D. J. Connelly F. M. (2000). Narrative inquiry: experience and story in qualitative research. San Francisco, CA: Jossey-Bass Publishers.

[B2] Connelly F. M. Clandinin D. J. (2006). “Narrative inquiry,” in Handbook of complimentary methods in education research. Editors Green J. L. Camili G. Elmore P. (Mahwah, NJ: Lawrence Erlbaum), 477–487.

[B3] Corbin C. Rolls J. A. (1996). The centre of the world at the edge of a continent: cultural studies of Cape Breton Island. Sydney, Australia: UCCB Press.

[B4] Dewey J. (1986). Experience and education. Educ. Forum.. 50 (3), 241–252. 10.1080/00131728609335764

[B5] Donatelli C. Murray C. Lionais D. Nicholson M. (2017). Our practice has had to change because of this: professional perceptions of long distance commuting in Atlantic Canada. The Extractive Industries and Society.. 4 (3), 606–613. 10.1016/j.exis.2017.05.003

[B6] Dorow S. Mandizadza S. (2018). Gendered circuits of care in the mobility regime of Alberta’s oil sands. Gend. Place Cult.. 25 (8), 1241–1256. 10.1080/0966369x.2018.1425287

[B7] Grzywacz J. G. Quandt S. A. Early J. Tapia J. Graham C. N. Arcury T. A. (2006). Leaving family for work: ambivalence and mental health among Mexican migrant farmworker men. J. Immigr. Minority Health.. 8 (1), 85–97. 10.1007/s10903-006-6344-7 19835002

[B8] Hall B. J. Pangan C. A. C. Chan E. W. W. Huang R. L. (2019). The effect of discrimination on depression and anxiety symptoms and the buffering role of social capital among female domestic workers in Macao, China. Psychiatr. Res.. 271, 200–207. 10.1016/j.psychres.2018.11.050 30500710

[B9] Hardy V. Lovei M. Patterson M. (2018). Recent trends in Canada’s labour market: a rising tide or a passing wave? Available at: https://www150.statcan.gc.ca/n1/pub/71-222-x/71-222-x2018001-eng.htm (Accessed June 9, 2020).

[B10] Haugen M. S. Brandth B. (2015). When farm couples break up: gendered moralities, gossip and the fear of stigmatisation in rural communities. Sociol. Rural.. 55 (2), 227–242. 10.1111/soru.12065

[B11] LeDrew H. M. Moores P. Read T. O’Regan-Hogan M. (2018). He’s here and he’s gone; he’s here and he’s gone . the experiences of new mothers in rural Newfoundland and Labrador, Canada, whose partners work away from home. Rural Rem. Health.. 18 (3), 4542. 10.22605/RRH4542 30208713

[B12] Leipert B. D. George J. A. (2008). Determinants of rural women’s health: a qualitative study in Southwest Ontario. J. Rural Health.. 24 (2), 210–218. 10.1111/j.1748-0361.2008.00160.x 18397458

[B13] Lionais D. Murray C. Donatelli C. (2020). Dependence on interprovincial migrant labour in Atlantic Canadian communities: the role of the Alberta economy. Societies. 10 (1), 11. 10.3390/soc10010011

[B14] Markey S. Ryser L. Halseth G. (2015). “We’re in this all together”: community impacts of long-distance labour commuting. Rural Soc.. 24 (2), 131–153. 10.1080/10371656.2015.1060717

[B15] McAuliffe M. Khadria B. Bauloz C. (2019). World migration report 2020. Geneva, Switzerland: IOM.

[B16] Mccoy H. V. Shehadeh N. Rubens M. (2016). Alcohol use and sexual risk behaviors in a migrant worker community. J. Immigr. Minority Health.. 18 (3), 561–567. 10.1007/s10903-015-0240-y PMC4696923 26123756

[B17] Meredith V. Rush P. Robinson E. (2014). “Fly-in fly-out workforce practices in Australia: the effects on children and family relationships.” text. Child Family Community Australia.. Available at: https://aifs.gov.au/cfca/publications/fly-fly-out-workforce-practices-australia-effects-children-and-fam/export (Accessed March 29, 2020).

[B18] Milbourne P. Kitchen L. (2014). Rural mobilities: connecting movement and fixity in rural places. J. Rural Stud.. 34, 326–336. 10.1016/j.jrurstud.2014.01.004

[B19] Morse C. E. Mudgett J. (2018). Happy to Be home: place-based attachments, family ties, and mobility among rural stayers. Prof. Geogr.. 70 (2), 261–269. 10.1080/00330124.2017.1365309

[B20] Murray C. F. (2014). “Coming and going: a narrative inquiry into women’s stories of a partner’s temporary interprovincial labor migration,” in Diss. Edmonton, Canada: University of Alberta.

[B21] Murray C. (2017). “Together yet living apart’: women left behind due to labour migration,” inCanadian community as partner: theory and multidisciplinary practice., Editor Robinson Vollman A. Anderson E. T. McFarlane J. (Philadelphia, PA: Wolters Kluwer), 327–333.

[B22] Neil K. C. Neis B. (2020). “Mobility has always been a part of my life”: work-related mobility and families in Canada. Can. Stud. Popul.. 47 (1), 111–118. 10.1007/s42650-020-00020-0

[B23] Nova Scotia Finance and Treasury Board (2017). Nova Scotia population estimates by county—July 1, 2016. Available at: https://novascotia.ca/finance/statistics/archive_news.asp?id=12629&dg=&df=&dto=0&dti=3 (Accessed June 9, 2020).

[B24] Parreñas R. S. (2000). Migrant filipina domestic workers and the international division of reproductive labor. Gend. Soc.. 14 (4), 560–580. 10.1177/089124300014004005

[B25] Parreñas R. S. (2001). Mothering from a distance: emotions, gender, and intergenerational relations in Filipino transnational families. Fem. Stud.. 27 (2), 361–390. 10.2307/3178765

[B26] P.E.I. Statistics Bureau (2019). Prince Edward Island population report. Available at: https://www.princeedwardisland.ca/sites/default/files/publications/pt_pop_rep_1.pdf (Accessed June 9, 2020).

[B27] Prince Edward Island Department of Finance (2019). Prince Edward Island 45th statistical review 2018. Available at: https://www.princeedwardisland.ca/sites/default/files/publications/web_2018_stat_review.pdf (Accessed June 9, 2020).

[B28] Scannell L. Gifford R. (2017). The experienced psychological benefits of place attachment. J. Environ. Psychol.. 51, 256–269. 10.1016/j.jenvp.2017.04.001

[B29] Skeldon R. (2012). Going round in circles: circular migration, poverty alleviation and marginality. Int. Migrat.. 50 (3), 43–60. 10.1111/j.1468-2435.2012.00751.x

[B30] Statistics Canada (2015). Canada goes urban. Available at: https://www150.statcan.gc.ca/n1/pub/11-630-x/11-630-x2015004-eng.htm (Accessed June 9, 2020).

[B31] Statistics Canada (2018). Canada at a glance 2018. Available at: https://www150.statcan.gc.ca/n1/en/pub/12-581-x/12-581-x2018000-eng.pdf?st=NXsMvLGh (Accessed June 9, 2020).

[B32] Statistics Canada (2019). Annual demographic estimates: subprovincial areas, July 1, 2018. Available at: https://www150.statcan.gc.ca/n1/pub/91-214-x/91-214-x2019001-eng.pdf (Accessed June 9, 2020).

[B33] Statistics Canada (2020). Labour force characteristics by economic region, three-month moving average, unadjusted for seasonality. Available at: https://www150.statcan.gc.ca/t1/tbl1/en/cv!recreate-nonTraduit.action?pid=1410029302 (Accessed June 9, 2020).

[B34] Taylor J. C. Graetz Simmonds J. (2009). Family stress and coping in the fly-in fly-out workforce. Aust. Comm. Psyc.. 21 (2), 23–36.

[B35] Torkington A. M. Larkins S. Sen Gupta T. (2011). The psychosocial impacts of fly-in fly-out and drive-in drive-out mining on mining employees: a qualitative study. Aust. J. Rural Health.. 19 (3), 135–141. 10.1111/j.1440-1584.2011.01205.x 21605226

[B36] Vodden K. Hall H. (2016). Long distance commuting in the mining and oil and gas sectors: implications for rural regions. The Extractive Industries and Society.. 3 (3), 577–583. 10.1016/j.exis.2016.07.001

[B37] Wathen C. N. Harris R. M. (2007). “I try to take care of it myself.” how rural women search for health information. Qual. Health Res.. 17 (5), 639–651. 10.1177/1049732307301236 17478646

[B38] Wray D. (2012). “Daddy lives at the airport”: the consequences of economically driven separation on family life in the post-industrial mining communities of Cape Breton. Empl. Responsib. Rights J.. 24 (2), 147–158. 10.1007/s10672-012-9196-4

